# A Continuum Model for Metabolic Gas Exchange in Pear Fruit

**DOI:** 10.1371/journal.pcbi.1000023

**Published:** 2008-03-07

**Authors:** Q. Tri Ho, Pieter Verboven, Bert E. Verlinden, Jeroen Lammertyn, Stefan Vandewalle, Bart M. Nicolaï

**Affiliations:** 1 BIOSYST-MeBioS, Faculty of Bioscience Engineering, Katholieke Universiteit Leuven, Leuven, Belgium; 2 Scientific Computing Research Group, Computer Science Department, Katholieke Universiteit Leuven, Leuven, Belgium; Spanish Council for Scientific Research (IIM-CSIC), Spain

## Abstract

Exchange of O_2_ and CO_2_ of plants with their environment is essential for metabolic processes such as photosynthesis and respiration. In some fruits such as pears, which are typically stored under a controlled atmosphere with reduced O_2_ and increased CO_2_ levels to extend their commercial storage life, anoxia may occur, eventually leading to physiological disorders. In this manuscript we have developed a mathematical model to predict the internal gas concentrations, including permeation, diffusion, and respiration and fermentation kinetics. Pear fruit has been selected as a case study. The model has been used to perform in silico experiments to evaluate the effect of, for example, fruit size or ambient gas concentration on internal O_2_ and CO_2_ levels. The model incorporates the actual shape of the fruit and was solved using fluid dynamics software. Environmental conditions such as temperature and gas composition have a large effect on the internal distribution of oxygen and carbon dioxide in fruit. Also, the fruit size has a considerable effect on local metabolic gas concentrations; hence, depending on the size, local anaerobic conditions may result, which eventually may lead to physiological disorders. The model developed in this manuscript is to our knowledge the most comprehensive model to date to simulate gas exchange in plant tissue. It can be used to evaluate the effect of environmental stresses on fruit via in silico experiments and may lead to commercial applications involving long-term storage of fruit under controlled atmospheres.

## Author Summary

Respiration plays an important role in the overall metabolism of plants, and certainly is related to gas exchange of plants with the environment. In roots and bulky storage organs such as fruit and tubers, where the length of the diffusion path may be considerable, anoxic conditions may even occur. This is of particular importance in fruit, which are often stored under low-oxygen conditions to extend their storage life. In this manuscript, we have developed a new mathematical model to describe the gas transport and respiration kinetics in intact pear fruit. Michaelis-Menten kinetics was used to describe the respiration behavior of tissues. Diffusion was the main driving force for gas exchange. Differences in diffusion rates of the different gasses led to total pressure gradients that caused convective exchange as described by Darcy's law. The model incorporates the actual shape of the fruit and was solved using fluid dynamics software. It is a first step towards a multiscale model that addresses all spatial scales relevant to gas transport. These findings can be used to evaluate the effect of environmental stresses on fruit via in silico experiments and may lead to commercial solutions for long-term storage of fruit under controlled atmospheres.

## Introduction

Exchange of O_2_ and CO_2_ of plants with their environment is essential for metabolic processes such as photosynthesis and respiration. Plants do not have specialised systems for gas exchange but rely on apertures in the epidermis such as stomata and lenticels and the intercellular air space within the tissue [1]. Also, in metabolically active organs such as leaves, the diffusion path is usually very short, thus facilitating gas transport. O_2_ and CO_2_ gradients have, however, been observed in plant organs such as roots [2], tubers [3], stems [4], inflorescences [5], seeds [6] and fruit [7]. In roots and bulky storage organs such as fruit and tubers, where the length of the diffusion path may be considerable, anoxic conditions may even occur. Geigenberger et al. [3] observed internal O_2_ concentrations below 5 kPa, causing partial inhibition of respiration, decrease in the cellular energy status, and partial inhibition of other energy-consuming processes. In some fruits such as pears, which are typically stored under a controlled atmosphere with reduced O_2_ and increased CO_2_ levels to extend their commercial storage life, anoxia may even occur, eventually leading to cell death and loss of the product [8]. Similar atmosphere conditions, however, do not seem to affect other fruit such as apples appreciably [9],[10]. While it is likely that this is related to differences in concentration gradients resulting from differences in tissue diffusivity and respiratory activity, there is little information about such gas gradients in fruit in the literature. Such knowledge would be, nevertheless, very valuable both to understand gas exchange in plant tissue but also to guide commercial storage practices, since disorders under controlled atmosphere related to fermentation are a prime cause of concern [10]–[13].

Microsensors have been used to measure oxygen concentrations in stem transects and phloem exudate of intact *Ricinus communis* plants [4], roots [14], and fruit [15],[16]. However, no matter how small the electrodes, insertion in fruit tissue causes damage that may result in measurement artefacts. Morison et al. [17] used chlorophyll fluorescence imaging to investigate CO_2_ diffusion into leaves; while this technique provides spatial information it obviously can only be used when there is an active photosynthetic system, which is not the case in fruit parenchyma cells. Biochemical measurements of indicators of anaerobiosis in roots such as acetaldehye, ethanol and alcohohol dehydrogenase have been carried in roots [18] and in fruit [19] but are indirect and do not provide quantitative data on gas concentrations.

As there is, to date, no good method to measure *in vivo* internal gas concentrations in fruit, a mathematical modelling approach would provide an alternative to predict the internal gas concentrations. Also, once validated such a model could be used conveniently to perform *in silico* experiments to evaluate the effect of, e.g., fruit size or ambient gas concentration on internal O_2_ and CO_2_ levels without the need for extra experimental effort. Denison [20] developed a reaction-diffusion model for oxygen diffusion and respiration in legume root nodules and found large effects of flooding of the intercellular space on the O_2_ permeability. Aalto and Juurola [21] constructed a three-dimensional model of CO_2_ transport in leafs and implemented it into a computational fluid dynamics code. The model accounts for the actual 3D microstructure of a leaf. The authors used the model to investigate the effect of stomatal opening, photosynthetic capacity, temperature and increased ambient CO_2_ levels on total CO_2_ flux.

Gas exchange in fruit and other bulky storage organs was first modelled macroscopically with Fick's first law as a diffusion process, which is driven by concentration gradients [22]–[24]. The concentration gradients appear because of consumption of O_2_ and production of CO_2_. However, Fick's first law is not capable of describing spatial gas concentration gradients. Several authors [7],[25],[26] therefore, developed reaction-diffusion models to describe the exchange of O_2_ and CO_2_ inside fruit of different plant species. Diffusion properties of fruit tissue were determined by measuring gas exchange through small tissue samples [27]–[32]. The results showed that the CO_2_ diffusivity in apple and pear tissue was much higher than the O_2_ diffusivity. However, as this may cause the outflow of CO_2_ to be larger than the inflow of O_2_, a pressure difference between the inside of the fruit and the external atmosphere may develop. Hence, besides gas diffusion driven by concentration gradients, gas exchange in the fruit may occur by permeation due to pressure gradients in the fruit tissues. The aforementioned models are not capable of describing this effect, and Ho et al. [31] therefore developed a permeation-diffusion-reaction model for describing O_2_, CO_2_ and N_2_ exchange in pear fruit which does take into account permeation. While this model was used successfully to simulate transport properties in simple disk-shape geometries, it cannot be used to study gas transport in intact pear fruit for the following reasons:

- A pear has a complicated shape which cannot be approximated well by a generic geometry such as a slab, sphere or cylinder.
- While the model has been used to evaluate the anisotropy of the gas transport properties of the tissue, its actual formulation is isotropic.
- Respiration is considered to be constant while it is known to depend heavily on the gas concentration [33]; this is actually the basis for the success of controlled atmosphere storage of pome fruit, and any model to be used to understand gas transport in fruit tissue during such storage procedures should incorporate this behaviour.
- The model cannot predict carbon dioxide production through fermentation at low O_2_ concentrations which actually puts a biochemical limit on how low the O_2_ partial pressure can be reduced in commercial postharvest storage procedures [8],[19].

The objective of this manuscript was, therefore, to extend this model to account for O_2_ and CO_2_ dependent respiration and fermentation processes. The resulting nonlinear model will be numerically solved for an actual pear geometry and validated using measurements on intact pears. *In silico* experiments will be carried out to study the effect of shape, size, temperature and storage atmosphere composition on macroscopic gas exchange in intact fruit.

## Results

### Overview of the continuum-model for exchange of metabolic gasses

A permeation-diffusion-reaction model was constructed to describe exchange of the three major gas atmospheric gases O_2_, CO_2_, and N_2_ in pear fruit based on the model described in [31]. The model assumes that gas exchange can be modeled by the lumped properties of the different fruit tissues. Gas exchange properties were independently and experimentally determined. The measurement protocols of gas exchange properties of pear epidermis and cortex tissue were described by Ho et al. [30],[31]. The driving force for gas exchange was mainly diffusion. Differences in diffusion rates of the different gasses led to total pressure gradients that caused convective exchange as described by Darcy's law [31]. Gas exchange was coupled with the respiration kinetics of fruit tissue. A non-competitive inhibition type of respiration kinetics was applied for O_2_ consumption and CO_2_ production [33],[34]. Kinetic parameters were estimated by means of respiration experiments. The permeation-diffusion-reaction model was applied to the axi-symmetric geometry of pear with variations of gas concentrations in the radial (*r*) and vertical axis (*z*) (see Materials and Methods). The full set of model variables is listed in Table 1. Both steady and transient simulations were carried out with different external conditions to study the spatial distribution of metabolic gasses in intact pears of different shapes and sizes.

**Table 1:** Respiration parameters of pear tissue and their 95% confidence interval.

Parameters	Units	Estimated Values Based on Tissue Measurement
*V_m,O2,tissue_* (20°C)	mol m^−3^ s^−1^	(2.39±0.14)×10^−4^
*V_m,O2,skin_*(20°C)	mol m^−3^ s^−1^	(8.31±2.52)×10^−4^
*V_m,f,CO2,tissue_*(20°C)	mol m^−3^ s^−1^	(1.61±0.13)×10^−4^
*V_m,f,CO2,skin_*(20°C)	mol m^−3^ s^−1^	(4.02±1.33)×10^−4^
*E_a,VmO2_*	kJ mol^−1^	80.2±12.3
*E_a,VmfCO2_*	kJ mol^−1^	56.7±13.3
*K_m,O2_*	kPa	1.00±0.23
*K_mn,CO2_*	kPa	66.4±21.3
*K_m,f,O2_*	kPa	0.28±0.14
*r_q,ox_*		0.97±0.04
*D_O2,skin_*	m^2^ s^−1^	(1.86±0.70)×10^−10^ a
*D_CO2,skin_*	m^2^ s^−1^	(5.06±3.3)×10^−10^ a
*D_N2,skin_*	m^2^ s^−1^	(1.06±0.29)×10^−10^ b
*K_skin_*	m^2^	(2.17±1.43)×10^−19^ b
*D_O2,r_*	m^2^ s^−1^	(2.8±1.59)×10^−10^ b
*D_CO2,r_*	m^2^ s^−1^	(2.32±0.41)×10^−9^ a
*D_N2,r_*	m^2^ s^−1^	(2.67±1.62)×10^−10^ b
*K_r_*	m^2^	(2.35±1.81)×10^−19^ b
*D_O2,z_*	m^2^ s^−1^	(1.10±0.40)×10^−9^ b
*D_CO2,z_*	m^2^ s^−1^	(6.97±2.19)×10^−9^ a
*D_N2,z_*	m^2^ s^−1^	(1.06±0.66)×10^−9^ b
*K_z_*	m^2^	(4.51±1.66)×10^−17^ b

The parameters were estimated by fitting predicted O_2_ consumption and CO_2_ production rates of pear tissue samples based on the respiration submodel (Equations 1–4) to measured data by means of a least squares procedure. Diffusion parameters were taken from Ho et al. [30],[31]. ±95% confidence limits. Indices *skin*, *r* and *z* refer to the position of the skin, along the radial direction and along the vertical axis of pear, respectively.

a Values measured by Ho et al. [30].

b Values measured by Ho et al. [31].

### Respiration of pear tissue

**Figure 1:**
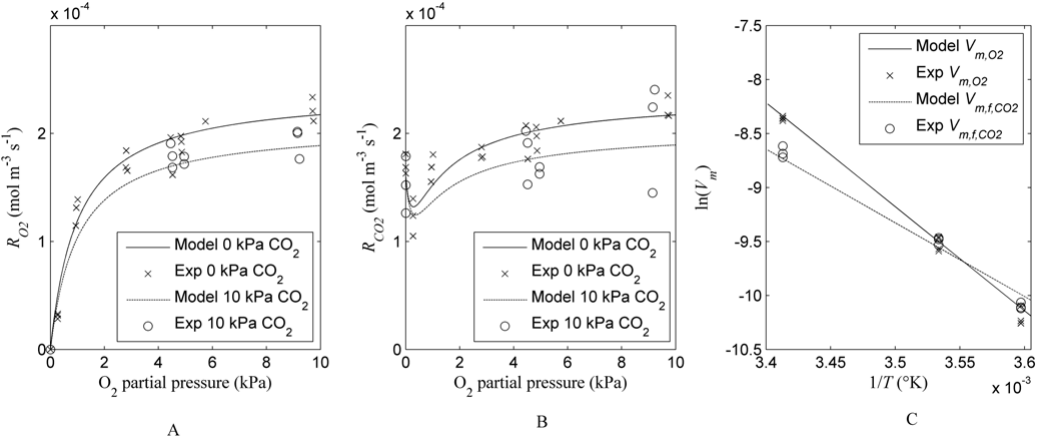
Respiration of tissue. (A) and (B) O_2_ consumption and CO_2_ production rate in pear tissue disks at 20°C. (C) the Arrhenius plot of maximal O_2_ consumption and CO_2_ production rate of tissue at different temperatures. Symbols denote measurement and the lines denote fitted model equation.

The non-competitive inhibition model for O_2_ consumption and CO_2_ production described the measured values well with an adjusted *R* ^2^ of 0.94 (Figure 1), indicating that the model was able to explain 94% of the total variability of the data after correction for the number of degrees of freedom. The correlation coefficients were all smaller than 0.71, suggesting that the model was not overparameterised. Note that because of this correlation between the parameters the confidence intervals are likely to be underestimated and are larger in reality.

The estimated parameters for *V*_m,O2_ and *V_m,f,CO2_* of cortex tissue were (2.39±0.14)×10^−4^ mol m^−3^ s^−1^ and (1.61±0.13)×10^−4^ mol m^−3^ s^−1^, respectively (Table 1). The confidence interval is dominated by the biological variability. *K_m,O2_*, a measure for the saturation of respiration with respect to O_2_ was relative small and equal to (1.00±0.23) kPa. A significant but low inhibition effect of CO_2_ on O_2_ consumption of pear cortex tissue was found (*K_mn,CO2_* = 66.4±21.3 kPa). The respiration quotient *r_q,ox_* was 0.97±0.04 and showed that the O_2_ consumption was about the same as the oxidative CO_2_ production. The value *K_m,f,O2_* is a measure of the extent to which fermentation can be inhibited by O_2_. The estimated value of 0.28±0.14 kPa implies that fermentation was already inhibited at very low levels of O_2_ concentration. The accuracy of the estimated parameter is reflected in the standard errors of estimation. The high standard error of *K_m,f,O2_* is due to the limited amount of information available in the data on the inhibition of fermentation by O_2_ since in the experimental used set-up it was not possible to obtain very small values of O_2_ partial pressure could not be obtained. Temperature had a significant effect on the respiration of the pear cortex tissue with values of *E_a,Vm,O2_* and *E_a,Vmf,CO2_* equal to (80.2±12.3) kJ mol^−1^ and (56.7±13.3) kJ mol^−1^, respectively.

### Validation of the respiration diffusion model

#### Respiration of intact pear

The gas concentration profiles of O_2_ and CO_2_ in the jar as function of time as calculated (Equation 10) from experimentally measured respiration kinetics of intact pear [33] were compared with predictions from the permeation-diffusion-reaction model (Equations 7–9). At 1°C the correspondence between measured and predicted gas concentrations is perfect. With increasing temperature, an increasing deviation of the CO_2_ profile was found; predicted values were larger than those experimentally observed. As the CPU time to solve the model was considerable because of the nonlinearity of the respiration kinetics in combination with the required spatial resolution at the boundary of the fruit, it was not feasible to carry out a Monte Carlo analysis to construct confidence intervals of the model predictions.

**Figure 2:**
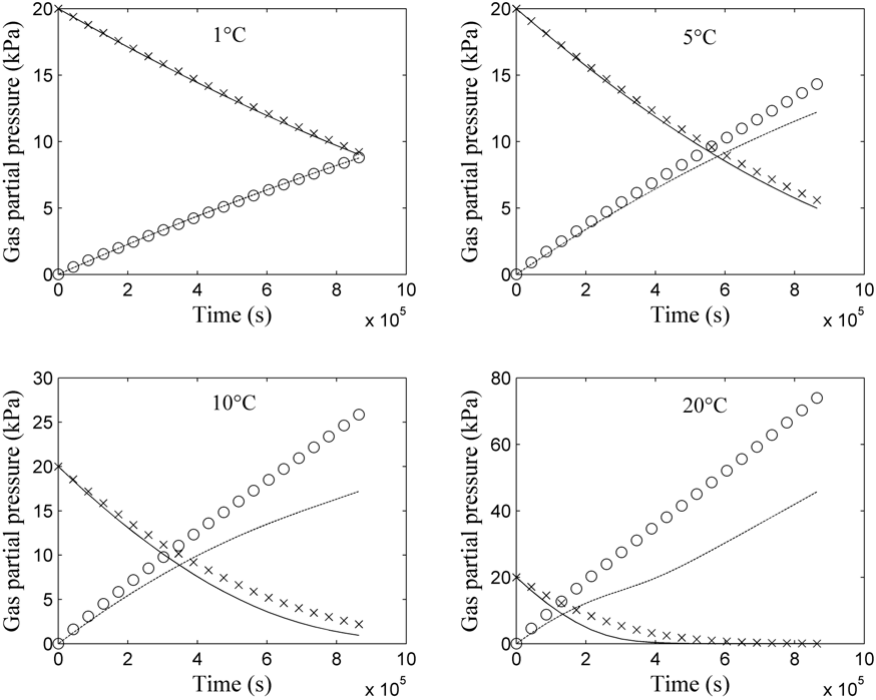
O_2_ and CO_2_ concentration as a function of time in closed jar at different temperatures (1, 5, 10 and 20°C). Solid lines (—) and dashed lines (- -) indicate the O_2_ and CO_2_ gas partial pressure in jar calculated by Equation 10 from the experimentally determined respiration kinetics [33]. The symbols (×) and (ο) indicate the O_2_ and CO_2_ gas partial pressure predicted by the permeation-diffusion-reaction model.

The parameters of the respiration kinetics of intact pear were also estimated from the simulated O_2_ and CO_2_ partial pressure profile in the jar as function of time (adjusted *R* ^2^ = 0.97). Their values are compared to the experimental values measured by Lammertyn et al. [33] in Table 2. There is clearly a good agreement between the two sets, except for *V_m,f,CO2_*. The latter discrepancy is believed to be the reason for the difference in CO_2_ profiles at higher temperature displayed in Figure 2.

**Table 2:** Respiration parameters of intact pear and their 95% confidence interval as determined by Lammertyn et al. [26] (column 3).

Parameters	Units	Literature Values	Computed from Permeation-Reaction-Diffusion Model
*V_m,O2,intact_* (20°C)	mol m^−3^ s^−1^	(2.13±0.24)×10^−4^	(2.37±0.27)×10^−4^
*V_m,f,CO2,intact_*(20°C)	mol m^−3^ s^−1^	(0.965±0.199)×10^−4^	(1.64±0.07)×10^−4^
*E_a,VmO2_*	kJ mol^−1^	64.6±4.7	67.6±2.5
*E_a,VmfCO2_*	kJ mol^−1^	58.5±8.9	61.4±2.5
*K_m,O2_*	kPa	6.2±0.9	5.3±1.7
*K_mn,CO2_*	kPa	70.7±21.6	92.3±132.5
*K_m,f,O2_*	kPa	0.69±0.17	0.86±1.71
*r_q,ox_*		0.76±0.03	0.79±0.05

The values computed from the permeation-reaction-diffusion model are listed in column 4. ±denotes 95% confidence limits.

#### Composition beneath the epidermis

In this validation, the gas atmosphere just beneath the epidermis at different external atmosphere and temperature were simulated at steady state and compared to the experimental data reported by Lammertyn et al. [26]. The results are shown in Table 3.

**Table 3:** Simulated and experimental subepidermal gas concentrations skin of pears for different ambient conditions.

Condition		Ambient Atmosphere					
		20.8% O2, 0% CO2		20.8% O2, 0% CO2		0% O2, 0% CO2	
		T = 20°C		T = 1°C		T = 20°C	
		O2 (kPa)	CO2 (kPa)	O2 (kPa)	CO2 (kPa)	O2 (kPa)	CO2 (kPa)
Measurement		14.7^a^	4.8^a^	20.7^a^	0.6^a^	0.2^a^	4^a^
σ*_experiment_*		1.0^a^	1.1^a^	0.2^a^	0.27^a^	0.071^a^	0.36^a^
Model	Surface	20.8	0	20.8	0	0	0
	Mean	14	4.7	19.3	0.59	0	4.4
	Subepidermal	9.7	7.7	18.6	0.97	0	7.1

The concentration at the outer surface of the fruit, just beneath the epidermis and the mean of both is reported. There is a good correspondence between the mean predicted concentrations and the measured ones. σ denotes the standard deviation.

a Data from Lammertyn et al. [26].

A good agreement (±1.4 kPa) was found between the measured and predicted values of the mean gas concentration. The results in Table 3 suggest that the permeation-diffusion-reaction model with adapted parameters is suited for predictions of gas exchange of intact fruit at the macroscopic level.

### Effect of permeation of the gas exchange in the whole fruit

**Figure 3:**
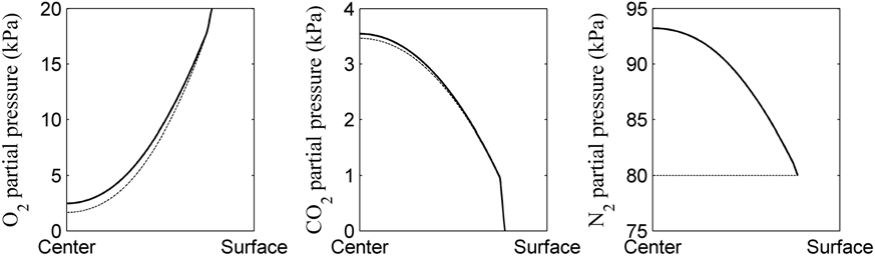
Effect of permeation on gas concentration distribution from the center to the surface along the radial direction. The simulation was carried out at 1°C, 20 kPa O_2_, 0 kPa CO_2_ at the ambient atmosphere. Solid lines (—) and dashed lines (- -) indicate the model with and without permeation, respectively.

To study the effect of permeation on gas exchange in whole fruit, Equations 7–9 were solved without and with permeation taken into account. Both simulations were done at 20 kPa O_2_ and 0 kPa CO_2_ as external conditions, both at 1°C. There was a significant effect of the permeation on the simulated result (Figure 3). The O_2_ and CO_2_ gas partial pressure profiles along the radial direction from the center to the surface of the pear are shown in Figure 3. Both the internal O_2_ and CO_2_ partial pressure was higher when permeation was included. While diffusion is the main process for the gas exchange inside the pear, the permeation clearly affects the gas exchange profiles.

Because there was an effect of permeation on gas exchange inside the fruit, at a certain environment atmosphere, the N_2_ partial pressure inside the fruit is not the same as the N_2_ partial pressure of the environment. Due to the respiration of the tissue, the O_2_ gas partial pressure decreased from the surface to the center of the pear while CO_2_ decreased in the opposite direction. An increase of N_2_ from the surface to the center of the pear was found (Figure 3).

The absolute ratio of inward convective 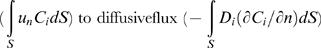 over the total pear surface was equal to 0.08, 0.0 and 1.0 for O_2_, CO_2_ and N_2_, respectively. While the convective flux is affected by the local magnitude of concentration, the diffusive flux is affected by the local gradient. The 0 value for CO_2_ is due to the fact that the concentration of CO_2_ is 0 at the surface. The value for O_2_ indicates that diffusion dominates the exchange, but permeation is not negligible. For N_2_ both mechanisms are equally important. This is confirmed by the profiles in Figure 3 showing the differences when permeation is included or not.

### Effect of geometry on local respiratory gas concentration

**Figure 4:**
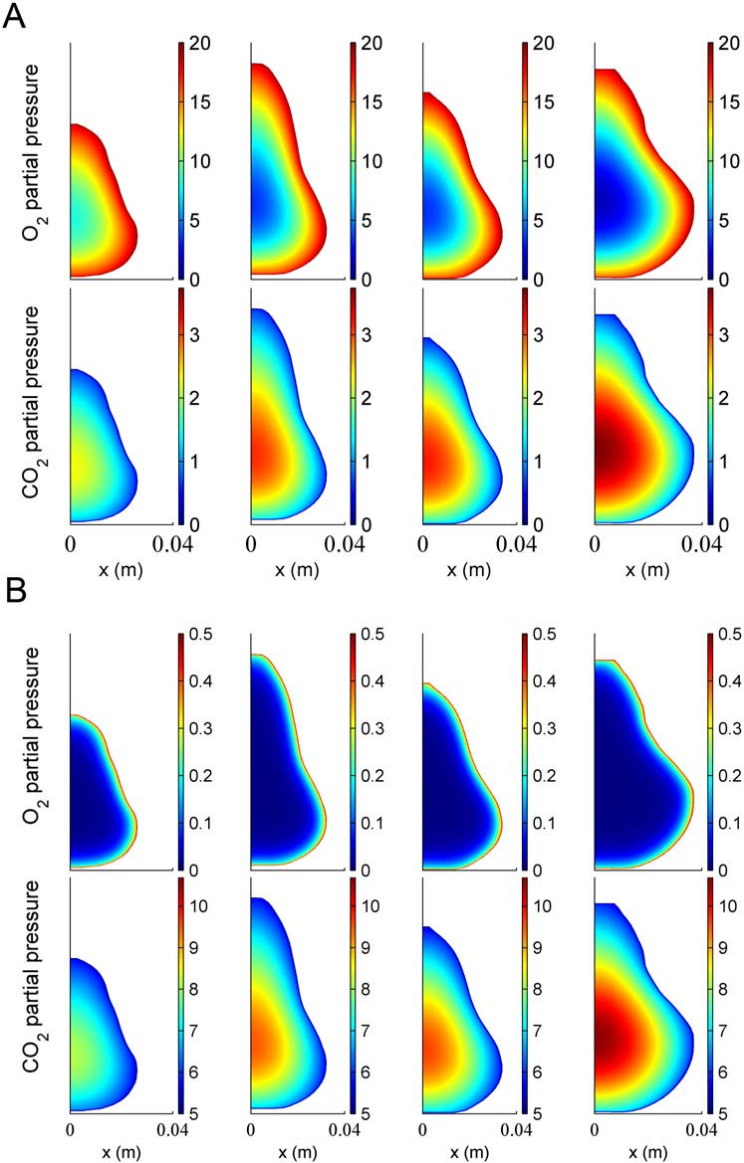
Steady state respiratory gas partial pressure distribution for different pear geometry. (A) −1°C, 20 kPa O_2_, 0 kPa CO_2_, 80 kPa N_2_. (B) −1°C, 0.5 kPa O_2_, 5 kPa CO_2_, 94.5 kPa N_2_. The latter conditions are known to induce the physiological disorder *core breakdown* in Conference pear [12].

Gas exchange was simulated for four pear shapes with different equatorial radii of 2.6; 3.2; 3.4 and 3.7 cm. In Figure 4A the respiratory gas partial pressure profiles in the four pears are shown for a storage gas atmosphere composition of 20 kPa O_2_, 0 kPa CO_2_ and 80 kPa N_2_ at −1°C (further called “regular air storage”). As expected, the gas concentration profiles are parallel to the boundary of the fruit. Due to the gas exchange barrier properties of the cortex and epidermis tissue, the partial pressures of O_2_ and CO_2_ in the center of the smallest pear (8.1 and 2.3 kPa) were significantly higher and lower, respectively, than those of the largest pear (1.1 and 3.74 kPa). The values of the other pears were in between these extremes. In Figure 4B the simulations were repeated for a storage gas atmosphere composition of 0.5 kPa O_2_, 5 kPa CO_2_ and 94.5 kPa N_2_ at −1°C (further called “core breakdown inducing controlled atmosphere storage”). The profiles are now very different from the previous ones. The partial pressures of O_2_ and CO_2_ in the center of the smallest pear were 1.23×10^−3^ and 8.14 kPa, respectively. In the largest pear they were 1.14×10^−3^ and 10.6 kPa, respectively.

## Discussion

### Permeation-diffusion-reaction model

Early models for gas exchange in plant tissue were based on Fick's first law [22]–[24]. They rely on the assumption that the diffusion resistance of the cortex tissue is low compared to that of the skin, which would exclude the existence of a gas gradient in the cortex tissue. The corresponding O_2_ and CO_2_ diffusion coefficients are then measured by effusion experiments. However, the steep gas gradient in Figures 3 and 4 indicate that these models are not applicable to pear cv Conference. Further, in the effusion method the diffusion coefficients of O_2_ and CO_2_ are calculated from that of an inert gas using Graham's law. This law states that the rate of effusion of a gas is inversely proportional to the square root of its molecular mass and would imply that the ratio of CO_2_ to O_2_ diffusivity would be 0.85. However, Schotsmans et al. [28] have shown that this law does not hold for a complex matrix such as fruit tissue and leads to underprediction of the CO_2_ diffusivity. This is confirmed by our data (Table 1), which suggest that the CO_2_ diffusivity of the skin (5.06×10^−10^ m^2^/s) is 2.7 times higher than that of O_2_ (1.86×10^−10^ m^2^/s). This is probably due to the larger solubility of CO_2_ in water than that of O_2_; while O_2_ would be transported mostly through the apoplast, CO_2_ would also diffuse through the cytoplasm.

More advanced reaction-diffusion models describing O_2_ and CO_2_ exchange in fruit have been reported in the literature [7],[25],[26],[35]. Mannapperuma et al. [25] found values of 2.67×10^−9^ m^2^ s^−1^ and 3.28×10^−9^ m^2^ s^−1^ for the O_2_ and CO_2_ diffusivity in ‘Golden Delicious’ apple tissue, which is larger than the values reported here (Table 1). This might be explained by the larger porosity of apple compared to that of pear. Schotsmans et al. [28] found values for the O_2_ diffusivity of skin (3.3×10^−10^ m^2^ s^−1^) and cortex tissue (4.3×10^−10^ m^2^ s^−1^) after 3 months of storage, which were comparable those reported here (Table 1). Further, they reported CO_2_ diffusivity values of 4.3×10^−10^ m^2^ s^−1^ and 1.73×10^−9^ m^2^ s^−1^ m^2^ s^−1^ for skin and cortex, respectively, which also correspond well with the values found here. Likewise, Lammertyn et al. [26],[27] found O_2_ diffusivity values of 2.84×10^−10^ m^2^ s^−1^ and 1.71×10^−9^ m^2^ s^−1^, and CO_2_ diffusivity values of 9.11×10^−10^ m^2^ s^−1^ and 1.95×10^−8^ m^2^ s^−1^ for skin and cortex respectively. It is not clear why the CO_2_ diffusivity of cortex found by Lammertyn et al. [26] is about ten times higher than that reported here. Note that in our model a distinction was made between the diffusivity in the axial and radial direction to account for the larger diffusivity in the axial direction due to vascular bundles which run from the stem to the calyx. The higher diffusivity in the axial direction compared to that along the radial direction is probably due to the fact that vascular bundles may be not fully filled with sap during storage of the fruit. It is, therefore, well possible that the vascular bundles along the axis of the pear indeed facilitate gas exchange. Moreover, the orientation of the cells along the vertical axis could be different from that of cells in the radial direction, and further difference in gas exchange properties may be due to enhanced interconnectivity of the gas intercellular space along the vertical axis compared to the radial direction [31]. Finally, while gas exchange properties might be affected by the developmental stage of the fruit through changes in tissue microstructure, it is interesting to note that Schotsmans et al. [28] did not find appreciable changes in gas diffusion properties of apple tissue during a period of seven weeks after harvest.

It should be emphasised that, because of the difference in diffusion coefficient, the produced CO_2_ leaves the fruit at higher rates than O_2_ is entering the fruit. This causes a pressure gradient inside the fruit. This pressure gradient initiates convective transport. Lammertyn et al. [26] found that the O_2_ partial pressure was under-predicted by this model and increased the O_2_ diffusivity parameter 3 times to improve the correspondence between measured and predicted O_2_ concentration. Such an adjustment was not required in the permeation-diffusion-reaction model presented here. The pressure gradient was alleviated in the model by a flux of N_2_ towards the center of the fruit.

While in the validation experiment at 1°C the correspondence between predicted and measured gas profiles was excellent, there was an increasing deviation for the CO_2_ profile with increasing temperature. As, in contrast to gas transport properties, the parameters of the respiration kinetics are highly dependent on temperature [33],[36],[37] it is likely that this mismatch can be related to the latter. We believe that this is due to the fact that fruit used for validation were different from those used for parameter estimation. In fact, the respiratory activity of pear depends on its maturity which can vary from season to season or batch to batch [16],[26]. Also, the preparation of disk samples for the respiration measurements might have caused an increase of the respiration rate [38],[39] due to an ethylene wound response [39],[40]. However, the available validation data are insufficiently informative to allow for re-estimating the respiration parameters. Novel experiments, possibly also providing data on internal gas concentrations, would be required.

Other models for gas exchange in plant organs have been developed. Denison [20] developed such a model for oxygen diffusion and respiration in legume root nodules while Parkhurst and Mott [41] described a reaction-diffusion model for CO_2_ assimilation in leaves. Aalto and Juurola [21] developed a model for CO_2_ exchange in leave parenchyma tissue. These models are based on the microscale geometry, and application to large organs such as fruit would require huge computer resources if possible at all. In contrast, the model developed here can be used well to predict gas exchange at the macroscale but does not provide detailed predictions of gas concentration at the microscale. We believe that microscale gas exchange models such as the ones developed by Denison [20] and Parkhurst and Mott [41] can be combined advantageously with macroscale models such as the one developed in this article. In such a multiscale approach, the macroscopic apparent diffusion coefficients can be estimated from *in silico* experiments using the microscale model. Multiscale modelling is an active area of materials engineering and physics [42] and has not been applied to plant physiology so far.

### Comparison of intact pear and pear tissue disk respiration

Tissue properties were measured using cylindrical tissue samples. The cutting process caused a film of juice at the cut surface of the samples which may fill up pores and, hence, affect the exchange properties. The cut surface was, therefore, always wiped off with cotton tissue. Further, the cell injury due to cutting leads to local enzymatic oxidation reactions which probably would not affect the apparent respiration rate considerably. However, cutting may possibly also illicit a stress response which might increase respiration. Such effects are difficult to quantify because there is currently no method available to measure in vivo gas exchange properties. However, it may explain some of the mismatches between measured and predicted respiration parameters of intact fruit.

Michelis-Menten kinetics are widely used to describe the relationship between the O_2_ concentration and the O_2_ consumption rate of whole intact fruit [43]. However, the O_2_ consumption rate is inhibited at high CO_2_ concentrations and the production of CO_2_ results from both oxidative and fermentative processes. As the Michelis-Menten model is not capable of describing such behaviour, it has been extended by various authors [34],[44],[45]. Such extended Michelis-Menten models can still be used as a semi-empirical model to describe the respiration characteristics of the whole intact fruit or vegetable. Here we have used such a model described by Equations 1–4 to describe both respiration of whole intact as well as tissue disks. However, the Michaelis-Menten constant *K_m,O2_* of the pear cortex tissue (1.00±0.23 kPa) was 6 times lower than for the intact pear (6.2±0.9 kPa) (Tables 1 and 2). This illustrates that the *K_m,O2_* value measured on the intact pears does not only contain information of the respiration but also about the macroscopic gas diffusion through pear cortex tissue and skin [35]. An even smaller value was also found for pear cell protoplast respiration (*K_m,O2_ = *3.0±0.3 µM corresponding to 0.18±0.03 kPa in the equilibrium gas phase) by Lammertyn et al. [33]. A similar result was also found for the inhibition of fermentation by CO_2_: the value for intact pear, *K_m,f,O2_*, was 0.69±0.17 kPa while it was 0.28±0.14 kPa for cortex tissue, or more than 2 times less. The high value of *K_mn,CO2_* (66.4±21.3 and 70.7±21.6 kPa for cortex tissue and intact pear, respectively) indicates that the inhibition effect of CO_2_ on respiration was small. This value is not exceptionally large, similar values were also found by Peppelenbos and Van't Leven [34] for Golden Delicious apple (64.1±49.8 kPa), Elstar apple (91±126 kPa) and asparagus (45.1±6.1 kPa). Smaller values were found for broccoli (11.5±2.3 kPa), mungbean sprouts (14.2±3.1 kPa) and cut chicory (13.5±4.8 kPa). There is no clear reason why there should be such a difference; in fact, relatively little is known about the effect of CO_2_ on the activity of respiratory enzymes.

The respiration quotient of pear cortex tissue (*r_q,ox_* = 0.97±0.04) was higher than that of intact pear (*r_q,ox_* = 0.76±0.03). We believe that the difference in *r_q,ox_* between cortex tissue and intact pear is due to the fact that CO_2_ has a high solubility in the water phase of fruit tissue (the CO_2_ capacity in the tissue α*_CO_*_2_ is equal to 0.948 at 20°C). As the estimation of *r_q,ox_* is essentially based on transient measurements of the gas profiles in the jar, it is well possible that CO_2_ is still accumulating in the intact pear because of the much larger internal gas exchange resistance compared with that of cortex tissue. Similar observations were made by Lammertyn et al. [26].

A good agreement was found between the *V_m,O2_* values of intact pear and pear cortex tissue, while the value of *V_m,O2_* of the skin was 3.5 times higher than the maximal respiration rate of the cortex tissue. A large respiration rate of the epidermis was also found by Lammertyn et al. [27] and Schotsmans et al. [28]. This may be due to the high density of the small cells in the epidermal region compared to the larger cells of the cortex region. The respiration of the skin as such might also be higher than that of cortex tissue.

The value of *V_m,f,CO2_* of the cortex tissue was 1.6 times higher than that of intact pear. This could be explained by the fact that the high solubility of CO_2_ in the water phase of fruit tissue leads to underestimation of the CO_2_ production of the intact pear. However, the value of *V_m,f,CO2_* for the tissue samples led to an over predicted value of the CO2 concentration in the validation experiment. Using the intact fruit value even resulted in better comparison with the validation set, but was outside the 95% confidence interval of the parameter. The reason for this mismatch is today unclear, but could be related to microscale balances of the different chemical forms of CO_2_ that are present in the intracellular liquid [46]. To resolve this issue, a microscale model of gas exchange and respiration in tissues that unravels the different mechanisms and species balances is required.

The O_2_ consumption rate of the intact pear was based on the O_2_ concentration decrease of the air atmosphere in the jar over a certain period of time. As the solubility of O_2_ in the water phase of fruit cortex tissue is low (α*_O_*_2_ is equal to 1.01×10^−1^ at 20°C), there was less effect on *V_m,O2_* of intact pear while the high solubility of CO_2_ in the water phase (α*_CO_*_2_ is equal to 9.49×10^−1^ at 20°C) of fruit cortex tissue could have significant affected *V_m,f,CO2_* and *r_q,ox_* of the intact pear.

### Effect of temperature on gas exchange

Temperature is the most important factor to control the fruit metabolism during storage. The influence of the temperature kinetic parameters can be described by the activation energy. Both the activation energy for O_2_ consumption (*E_a,Vm,O2_* = 80.2±12.3 kJ mol^−1^) and fermentative CO_2_ production (*E_a,Vmf,CO2_* = 56.7±13.3 kJ mol^−1^) of the cortex tissue were close to that of intact pear (*E_a,Vm,O2_* = 64.6±4.7 kJ mol^−1^ and *E_a,Vmf,CO2_* = 58.5±8.9 kJ mol^−1^) described by Lammertyn [35].

Temperature effects on respiration rate are well known, however, attempts to characterise temperature influence on tissue diffusion have not revealed substantial temperature effects [30]. Temperature had a small influence on the diffusion while tissue respiration showed a strong effect by temperature. A high concentration gradient was found at high temperatures. Therefore, storage temperatures should be low enough to have low respiration activity resulting in small gas gradients.

### Effect of pear size and storage atmosphere composition on gas exchange

Cytochrome c oxidase has been reported as the rate limiting enzyme in the respiration pathway [47]. Solomos obtained a value of 0.1 µM for the *K_m,O2_* for isolated cytochrome c oxidase for apple [48]. The calculated O_2_ concentration expressed in µM in the center of the smallest and largest pear under regular air storage was equal to 111 and 15 µM and, hence, much larger than this *K_m,O2_* value. Consequently, the O_2_ concentration will not be rate limiting and the respiratory pathway will be active. In contrast, under core breakdown inducing conditions the O_2_ concentration was equal to 1.69×10^−2^ and 1.56×10^−3^ µM, well below the cytochrome c oxidase *K_m,O2_* value. Under such conditions, and particularly in the large pear, the respiratory pathway would be blocked and fermentation is likely to occur. The latter storage conditions are in fact known to cause the physiological disorder core breakdown in pear, and large fruit are known to be much more susceptible to this disorder than small fruit [12]. These results show that the model developed in this article helps in explaining the occurrence of controlled atmosphere related physiological storage disorders in pear. This is a major step forward in understanding the biophysical processes underlying physiological disorders compared to statistical models such as the one developed by Lammertyn et al. [12].

### Conclusions

A permeation-diffusion-reaction model was developed to study gas exchange of intact pear at the macroscale level. The model accounts for both diffusion and pressure driven exchange of these gasses and incorporates anisotropic transport properties. O_2_ depletion and CO_2_ production because of respiration were modelled by means of Michaëlis Menten kinetics which were modified to account for O_2_ and CO_2_ inhibition effects. As the pear shape cannot be approximated by a generic geometry such as slab, sphere or cylinder, a computer vision system was used to reconstruct the actual geometry of the pear and the model equations were discretised over this geometry. The model was validated successfully under steady and transient conditions at 1°C; there was an increasing deviation for the CO_2_ profile with increasing temperature, probably due to season or batch effects on the parameters of the respiration kinetics. The model structure is generic: application of the model to other fleshy fruit is straightforward but the model parameters and the fruit geometry need to be measured.

Based on an *in silico* study it was found that considerable gradients of metabolic gases may exist in fruit, hereby invalidating earlier models in which it was assumed that gas transport could be lumped. Higher values of the Michaëlis-Menten parameters of the respiration of intact fruit compared to those of cortex tissue could be attributed to the gas exchange barrier function of fruit tissue. The larger the fruit, the lower and higher the O_2_ and CO_2_ partial pressures in the fruit center, respectively, indicating a larger susceptibility to fermentation and storage disorders. Further, the large differences in apparent diffusion coefficient for O_2_ and CO_2_ result in an underpressure in the centre of the fruit which causes permeation gas transport.

However, diffusion remains the main mechanism of gas exchange. An *in silico* study revealed that, in contrast to small pears, in large pears and under extreme storage conditions the oxygen concentration can decrease well below the Michaëlis Menten constant for cytochrome c oxidase, the rate limiting enzyme of the respiration pathways. This most probably leads to fermentation and physiological disorders which have been observed under such conditions. For the first time a plausible and quantitative biophysical explanation is given for the well-known role of gas exchange in the development of physiological disorders in fruit such as core breakdown.

The model developed in this article is a first step towards a comprehensive model of gas exchange of pear fruit. Further advances require that the internal microstructure of the tissue is investigated to explain differences in gas exchange properties and to quantify the cellular and intercellular pathways for gas exchange. Also, the respiration and fermentation submodels are phenomenological and do not allow to evaluate the effect of internal gas concentrations on cellular metabolic fluxes. Such information would help to explain physiological disorders related to oxidative stresses such as typical browning patterns in pear tissue stored under hypoxic conditions. Mechanistic models incorporating more detailed knowledge of the respiratory and fermentative pathways are currently being developed in our group. Finally, the respiratory metabolism does change dramatically during maturation and ripening of climacteric fruit such as pear in response to ethylene biosynthesis. Hence, the parameters of the respiration and fermentation submodels are likely to change as well and need to be estimated for different development stages.

## Materials and Methods

### Fruit material

Pears (*Pyrus communis* cv. ‘Conference’) were harvested on September, 8th, 2004, at the pre-climacteric stage at the Fruitteeltcentrum (Rillaar, Belgium), cooled and stored according to commercial protocols for a period of 21 days at −0.5°C preceding CA storage (2.5 kPa O_2_, 0.7 kPa CO_2_ at −0.5°C) until they were used for the respiration experiments on tissue discs.

### Respiration models

A non-competitive inhibition model [34],[44],[45] was used to describe consumption of O_2_ by respiration as formulated by Equation 1:
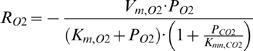
(1)with *V_m,O2_* (mol m^−3^ s^−1^) the maximum oxygen consumption rate, *P_O2_* (kPa) the O_2_ partial pressure, *P_CO2_* (kPa) the CO_2_ partial pressure, *K_m,O2_* (kPa) the Michaelis-Menten constant for O_2_ consumption, *K_mn,CO2_* (kPa) the Michaelis-Menten constant for non-competitive CO_2_ inhibition, and *R_O2_* (mol m^−3^ s^−1^) the O_2_ consumption rate of the sample.

The equation for production rate of CO_2_ consists of an oxidative respiration part and a fermentative part [43].
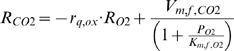
(2)with *V_m,f,CO2_* (mol m^−3^ s^−1^) the maximum fermentative CO_2_ production rate, *K_m,f,O2_* (kPa) the Michaelis-Menten constant of O_2_ inhibition on fermentative CO_2_ production, *r_q,ox_* the respiration quotient at high O_2_ partial pressure, and *R_CO2_* (mol m^−3^ s^−1^) the CO_2_ production rate of the sample.

The effect of temperature was described by Arrhenius' law [45].
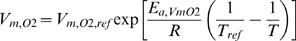
(3)

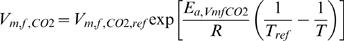
(4)with *V_m,O2,ref_* (mol m^−3^ s^−1^) and *V_m,f,CO2,ref_* (mol m^−3^ s^−1^) the maximal O_2_ consumption rate and maximal fermentative CO_2_ production rate at *T_ref_* = 293°K, respectively; *E_a,VmO2_* (kJ mol^−1^) the activation energies for O_2_ consumption; *E_a,VmfCO2_* (kJ mol^−1^) the activation energies for fermentative CO_2_ production; *T* (K) temperature; and *R* (8.314 J mol^−1^ K^−1^) the universal gas constant. It was assumed that the other parameters in Equations 1 and 2 do not depend on temperature [45].

Asymptotic confidence intervals were calculated from the asymptotic covariance matrix **C** of the parameters
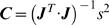
with **J** the Jacobian matrix with respect to the estimated parameters, and *s* ^2^ the mean squared error. The asymptotic (1−α)% confidence interval on the *i*-th parameter estimate *P_i_* was calculated as
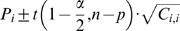
with *t* the Student t-distribution, *n* the number of measurements, *p* the number of parameters, and *C_i,i_* the *i*-th diagonal element of **C**.

### Permeation-diffusion-reaction model

The tissue structure of the fruit is considered to contain mainly two phases, the intra-cellular liquid phase of the cells and the air-filled intercellular space. Assuming local equilibrium at a certain concentration of the gas component *i* in the gas phase *C_i,g_* (mol m^−3^), the concentration of the compound in the liquid phase of fruit tissue normally follows Henry's law. If the tissue has a porosity *ε*, the volume-averaged concentration *C_i,tissue_* (mol m^−3^) of species *i* is then defined as:
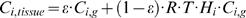
(5)with *H_i_* (mol m^−3^ kPa^−1^) Henry's constant of component *i* (*i* is O_2_, CO_2_ or N_2_), *R* the universal gas constant (8.314 J mol^−1^ K^−1^) and *T* (K) the temperature. From this definition, we derive the following expression for the gas capacity α*_i_* of the component *i* of the tissue
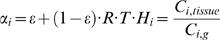
(6)Henry's constant was reported by Lide [49]. The porosity of Conference pear was determined by Schotsmans [50] from the density of intact fruit and juice, and was equal to 0.07.

A permeation-diffusion-reaction model was constructed describing the diffusion and permeation processes in pear tissue for the three major atmospheric gases O_2_, CO_2_ and N_2_. Equations for transport of O_2_, CO_2_ and N_2_ were established by Ho et al. [31],
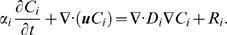
(7)At the boundary:
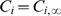
(8)with *D_i_* (m^2^ s^−1^) the apparent diffusion coefficient, **u** (m s^−1^) the apparent velocity vector, *R_i_* (mol m^−3^ s^−1^) the production term of the gas component *i* related to O_2_ consumption or CO_2_ production (Equations 1–4), ∇ (m^−1^) the gradient operator, and *t* (s) the time. The index ∞ refers to the gas concentration of the ambient atmosphere. The first term in Equation 7 represents the accumulation of gas *i*, the second term permeation transport driven by an overall pressure gradient, the third term molecular diffusion due to a partial pressure gradient, and the last term consumption or production of gas *i* because of respiration or fermentation. If, for example, oxygen is consumed in the center of the fruit, it creates a local partial pressure gradient which drives molecular diffusion. However, if the rates of transport of different gasses are different, overall pressure gradients may build up and cause permeation transport. Nguyen et al. [51] observed based on nuclear magnetic resonance imaging that the water concentration in pear fruit is almost uniform; gradients were restricted to a thin layer just beneath the surface. As a consequence, the water vapour pressure is also almost uniform within the fruit so that there was no need to model water vapour transport in the food.

It is important to note that the apparent diffusion coefficients are not physical properties as such but rather phenomenological parameters which depend on both the actual gas diffusion properties and fruit microstructure. Also, we have assumed that the size of the pores and channels connecting the pores are large compared to the mean free path of molecular motions which is typically 0.07 µm for N_2_ at 20°C and 10^5^ Pa [52]. As the structure of the intercellular space is essentially three-dimensional, appropriate visualisation techniques such as microfocus computer tomography are required to test this hypothesis [53].

Permeation through the barrier of tissue by the pressure gradient was described by Darcy's law [54]:
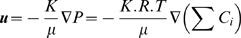
(9)with *K* (m^2^) the permeation coefficient; *P* (Pa) the pressure and µ (Pa.s) the viscosity of the gas. The relation between gas concentration and pressure was assumed to follow the ideal gas law (*P* = *CRT*).

### Measurement of tissue respiration

Respiration rate measurements on pear tissue were carried out at 20°C at 0, 0.5, 1, 3, 5, 10, 30 kPa O_2_ combined with 0 kPa of CO_2_ as described by Schotsmans et al. [28]. To study the inhibitory effect of CO_2_, respiration measurements were carried out at 0, 5, 10 and 30 kPa O_2_ in combination with 10 kPa CO_2_. For quantifying the effect of temperature on the respiration rate, measurements were carried out at 5, 10 and 20°C at 0 and 30 kPa O_2_ in combination with 0 kPa CO_2_. The samples were prepared in the same manner as the samples for the diffusion measurement as described by Ho et al. [30],[31]. Samples of cortex tissue were first cut with a professional slice cutter (EH 158-L, Graef, Germany). Subsequently, small cylinders with a diameter of 24 mm were cut with a cork borer. The thickness of the cortex tissue sample ranged from 1.5 to 2 mm. Skin samples (including both the epidermis and the hypodermis of the fruit) were cut and removed the flesh until a thickness of around 1mm was obtained. Samples of 55–65 pieces (∼50–60 g) were placed on metal meshes in 1.7 L glass jars. Three jars were connected in series and flushed with each gas mixture during 30 minutes. The jars were then closed and the initial gas mixture (O_2_, CO_2_ and N_2_) was measured with a gas chromatograph (Chrompack CP 2002, The Netherlands). Percentages of O_2_, CO_2_ and N_2_ were converted to partial pressures using the total pressure in the jar that was measured with a pressure sensor (DPI 142, GE Druck, Germany). The headspaces were analysed again after 17 h. The difference in gas partial pressure was converted to molar concentrations according to the ideal gas law, and from this the O_2_ consumption and CO_2_ production rates were calculated and expressed in mol per volume of sample (m^3^ fresh volume of sample) and per unit time (s).

All parameters of the respiration models (Equations 1–4 were estimated simultaneously by fitting Equations 1–4 to the experimental data using a non-linear least squares regression in MATLAB [The Mathworks]). The data on O_2_ consumption and CO_2_ production rates were pooled, and the same weight was attributed to both gases.

### Measurement of gas exchange coefficients

Permeation properties of pear epidermis and cortex tissue were determined by measuring the total pressure difference between two chambers separated by a tissue sample [31]. Both chambers were flushed with humidified N_2_ gas at 10 L/h. The pressure was adjusted so as to obtain a 6 kPa pressure difference between the measurement and flushing chamber. The inlet and outlet valves of one chamber were closed, and the decrease in pressure of this chamber was monitored for at least 4 h. The permeability was then estimated from this pressure drop using the procedure described by Ho et al. [31].

For the measurement of the diffusion properties, different gas concentrations were applied in both chambers [30] and the change of O_2_ and CO_2_ partial pressure in time was measured by means of fluorescent optical probes (Foxy-Resp and FCO_2_-R, Ocean Optics, Duiven, The Netherlands). The O_2_ and CO_2_ gradients were chosen in such a way that the resulting O_2_ and CO_2_ fluxes through the sample would compensate each other avoiding a total pressure difference between the two chambers. Pressure sensors (PMP 4070, GE Druck, Germany) monitored the pressure changes in each chamber during the measurements. The gas diffusion properties were then estimated from the gas concentration profiles as described by Ho et al. [30]. The N_2_ diffusivity was determined indirectly from the total pressure and the O_2_ partial gas pressure of the binary O_2_-N_2_ gas mixture [31]. The values used in this article were taken from Ho et al. [30].

### Pear geometry

Since the geometry of pear can be considered as axi-symmetric, there are variations of gas concentrations in the radial direction (*r*) and vertical axis (*z*) only and not in the angular direction. Therefore, the problem can be solved in two dimensions in the *r-z* plane instead of using a full three-dimensional model. This can save considerable memory and computation time resources.

**Figure 5:**
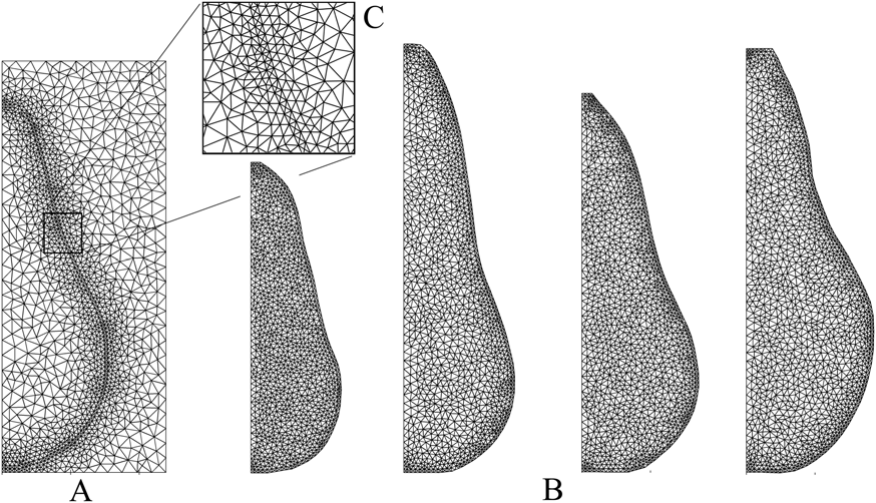
Geometric model and finite element mesh of pears. (A) Pear in jar. (B) Pears with different shapes. (C) An inset of the triangular mesh of the small part.

The two-dimensional shape of the fruit was constructed with a machine vision system for shape description [55]. An intact pear was put on a rotation table with a computer controlled stepper motor (Apollo, C-630.32, Physik Instrumentc GmbH, German) and pictures were taken with a CCD color digital video camera (DFW-VL500, Sony, Japan) along the fruit equator. The geometrical model of the pear was reconstructed using image processing software written in MATLAB. The image was segmented in object and background using an automatic threshold on the saturation value of the color information, and the contour of the object was extracted from the image. Subsequently, a cubic spline (smooth polygonal approximation) was fitted on the contour. In every node the normal vector to the contour was calculated, and the skin was defined as to have a thickness of 1 mm along this normal vector. The epidermis tissue (skin) was created by shrinking the contour along this normal vector until a skin thickness of 1 mm was obtained in every node. Note that skin here includes both the epidermis and the hypodermis–a relatively tightly packed diffuse layer of cells located in between the epidermis and cortex tissue to mimic the skin sample of gas exchange and respiration measurement. The same skin thickness was assumed for estimating the gas exchange properties [30],[31]. The geometrical description based on contour information was transferred to the Femlab version 3.1. finite element program (Comsol AB, Stockholm) package, where a finite element mesh was generated on the pear geometry. An axisymmetric geometry model was created for the pear in the jar (Figure 5). In total, 5441 quadratic finite elements with triangular shape were used. Note that Conference pears typically do not have an empty core, there was no need to incorporate a hole in the finite element model.

To study the effect of pear shape on the local respiratory gas inside the fruit, four geometries were established representing pears of different equatorial radius (2.6; 3.2; 3.4 and 3.7 cm - Figure 5).

### Numerical solution

For gas exchange in intact fruit, Equations 1–9 were solved using the finite element method in Femlab version 3.1. (Comsol AB, Stockholm). Anisotropic gas exchange properties were applied in the radial (*r*) and vertical (*z*) direction. Since the model for consumption of O_2_ inside the fruit (Equation 1) does not exclude negative concentrations, numerical problems may occur when the oxygen concentration approaches zero, resulting in non-physical negative results. Therefore, two alternative approaches were introduced to solve this problem. The first method was based on modifying the respiration term to ensure that the rate of O_2_ consumption became zero when the O_2_ concentration approached zero. Another method was based on the exponential transformation of the O_2_ variable in the model equations in such a way that the solution is guaranteed to be positive (see Text S1). A good agreement was found for both two solutions (not shown).

### Model validation

#### Respiration of intact pear

The permeation-diffusion-reaction model was validated by comparing the overall respiration kinetics of intact fruit with measured data which were available in our group [33]. Hereto, an *in silico* experiment was carried out in which a pear was put into a closed jar. The time course of the concentration of metabolic gases *C_i,jar_* (mol m^−3^) is then given by
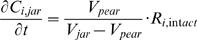
(10)with *V_pear_* (m^3^) the volume of pear; *V_jar_* (m^3^) the volume of jar; *R_i,intact_* (mol m^−3^ s^−1^) the respiration of the intact fruit due to O_2_ consumption or CO_2_ production. *R_i,intact_* was described by means of non-competitive inhibition kinetics according to Lammertyn et al. [33] who also estimated the corresponding model parameters from experimental data (Table 1). The calculated *C_i,jar_* were considered as experimental data and compared to model predictions. Gas exchange properties of tissue were taken from Ho et al. [30],[31] and the respiration parameters of tissue were reported in Table 1 and 2. To derive the initial gas concentration in the fruit, the steady state model equations were solved for 20 kPa O_2_ and 0 kPa CO_2,_ and for different temperatures (1, 5, 10, 20°C) to mimic the conditions of the actual respiration measurement.

#### Gas atmosphere beneath the epidermis

Subepidermal gas concentration measurements were available in our group [26]. These authors glued an airtight respiration chamber of small volume to the surface at the thickest part of the pear and sealed it completely from the ambient atmosphere. Subsequently, the pears were put in air flushed jars to equilibrate under their respective storage atmosphere. At the equilibrium condition (steady state), a gas sample of the respiration chambers was analysed with a micro-GC (Chrompack CP 2002, The Netherlands). The gas concentration just beneath the epidermis was also simulated using the permeation-diffusion-reaction model with three different external conditions: 20.8% O_2_ and 0% CO_2_ at 20°C; 0% O_2_ and 0% CO_2_ at 20°C; and finally 20.8% O_2_ and 0% CO_2_ at 1°C. The experimentally determined subepidermal gas concentrations were then compared with predicted values.

## Supporting Information

Text S1 Appendix: Numerical scheme for the permeation-diffusion-reaction model.
(0.04 MB DOC)
