# Supplementary material for: A Continuum Model for Metabolic Gas Exchange in Pear Fruit
Source: PLoS Comput Biol. 2008 Mar 7;4(3):e1000023. doi: 10.1371/journal.pcbi.1000023 (PMC2265468; doi:10.1371/journal.pcbi.1000023)
Supplement: Text S1 — Appendix: Numerical scheme for the permeation-diffusion-reaction model. (0.04 MB DOC) [file pcbi.1000023.s001.doc]

**APPENDIX Numerical scheme for the permeation-diffusion-reaction model**

1. To ensure that the O_2_ concentration cannot become negative due to O_2_ consumption ($R_{O2}\leq0$), the respiration term in the permeation-diffusion-reaction model was modified for the O_2_ and CO_2_ in the solution:

If $C_{O2,g}<0$ then $R_{O2}=0$ (11) and $R_{CO2}=V_{m,f,CO2}$ (12)

If $C_{O2,g}\geq0$ then $R_{O2}$ and $R_{CO2}$ are described by Eq. (1) and (2).

Analytically, there is no O_2_ consumption when O_2_ reaches zero. Therefore, the O_2_ concentration should never become negative. It is clear that when the O_2_ concentration is positive, the reaction terms are the same as the original model. The solution, therefore, will be physically consistent.

2. Transformation of the governing equation

An exponential transformation of the main variable was used to impose positive values for the O_2_ concentration:

$C_{O2,g}=\exp\left(U_{O2}\right)$ (13)

Hence, Eq. (7) for the O_2_ was transformed into

$\left(\exp\left(U_{O2}\right)\cdot\alpha_{O2}\right)\frac{\partial U_{O2}}{\partial t}+\nabla\cdot\left(\mathbf{u}\cdot\exp\left(U_{O2}\right)\right)=\nabla\cdot\left(D_{O2}\cdot\exp\left(U_{O2}\right)\right)\nabla U_{O2}+R_{O2}$ (14)

At the boundary $U_{O2,r}=\ln\left(C_{O2,\infty}\right)$ (15)

The permeation model (Eq. (9)) was transformed into

$\mathbf{u}=-\frac{kRT}{\mu}\nabla\left(\sum C_{i,g}\right)=-\frac{kRT}{\mu}\left(\exp\left(U_{O2}\right)\nabla U_{O2}+\nabla C_{CO2,g}+\nabla C_{N2,g}\right)$ (16)

Similarly, exponential transformation of the O_2_ concentration was applied in the respiration equations (1) and (2).
